# 推进血液重症监护病房建设，提高血液专科重症救治能力

**DOI:** 10.3760/cma.j.cn121090-20250922-00432

**Published:** 2025-11

**Authors:** 圣瑾 范, 海涛 李, 曦 张

**Affiliations:** 1 哈尔滨医科大学附属第一医院血液内科重症监护病房，哈尔滨 150001 Hematology Intensive Care Unit, the First Affiliated Hospital of Harbin Medical University, Harbin 150001, China; 2 陆军军医大学第二附属医院（新桥医院）血液病医学中心，血液生态与智慧细胞科学创新中心，中国人民解放军血液病中心，全军临床重点专科，重庆市临床重点专科，创伤与化学中毒全国重点实验室，重庆 400037 Medical Center of Hematology, the Second Affiliated Hospital of Army Medical University（Xinqiao Hospital）; Institute of Science Innovation for Blood Ecology and Intelligent Cells, PLA Center for Hematologic Diseases, PLA Key Clinical Specialty, Chongqing Municipal Key Clinical Specialty, State Key Laboratory of Trauma and Chemical Poisoning, Chongqing 400037, China

## Abstract

探讨建设血液重症监护病房（HCU）对于提升血液专科重症救治能力的重要性。本文介绍我国HCU的两种运行模式（ICU模式与pre-ICU模式）及实践经验，主张在血液病区内建设、完善呼吸循环监护与血液净化等设备，由接受重症培训的血液科医师主导，或通过标准化流程与综合ICU高效协作；对尚未达标的单位建议设抢救室并加强重症培训。HCU主要收治对象为血液病相关严重或致命性出血、重症感染与脓毒性休克、初诊急性髓系白血病/急性早幼粒细胞白血病（AML/APL）早期并发症、治疗相关并发症、进展迅速高致死性疾病及需器官支持或血液净化患者。强调病情量化评估与早期转入：应用国家早期预警评分（NEWS）、quick-序贯器官衰竭评分（SOFA）、SOFA/ΔSOFA、多国癌症支持治疗协会（MASCC）等工具分层，尽早实施脓毒症集束化治疗，优先经鼻高流量湿化氧疗（HFNC）改善氧合；在肾功能衰竭伴少尿或无尿、充血性心力衰竭、药物过量等指征下行连续性肾脏替代治疗（CRRT）；原发病治疗遵循“三级分层”和“观察-调整”策略，兼顾患者意愿。倡议具备条件的血液中心积极筹建HCU，探索诊疗新模式，提高血液专科重症救治能力。

## Abstract (en)

Establishing hematology care units（HCU）is essential for strengthening hematology-specific critical care capacity. This article outlines two HCU operational models in China—the ICU model and the pre-ICU model—and discusses practical insights from their implementation. It recommends establishing HCU within hematology wards, equipped for respiratory and hemodynamic monitoring and blood purification, and led by hematologists trained in critical care or by teams collaborating efficiently with general ICU through standardized workflows. For units not yet meeting these standards, establishing a resuscitation room and strengthening critical care training are advised. HCU primarily admit patients with severe or life-threatening bleeding due to hematologic diseases, severe infections or septic shock, early complications of newly diagnosed acute myeloid leukemia or acute promyelocytic leukemia（AML/APL）, treatment-related complications, rapidly progressive or highly lethal conditions, and those requiring organ support or blood purification. Emphasis is placed on quantitative assessment of disease severity and timely transfer to the HCU, using tools such as the National Early Warning Score, quick Sequential Organ Failure Assessment（qSOFA）, SOFA/ΔSOFA, and the Multinational Association of Supportive Care in Cancer risk index for patient stratification. Early implementation of sepsis bundles is emphasized. For organ support, high-flow nasal cannula oxygen therapy is prioritized to improve oxygenation. Continuous renal replacement therapy is performed when there are indications such as renal failure with oliguria or anuria, congestive heart failure, or drug overdose, etc. Treatment of the underlying hematologic disease follows a “three-tier stratification” and “watch-and-adjust” strategy, while respecting patient preferences. We encourage eligible hematology centers to actively establish HCU, explore new care models, and strengthen hematology-specific critical care capacity.

血液病患者，尤其是恶性血液肿瘤患者被视为脆弱群体，常伴有严重免疫功能缺陷，易发展为急危重症，且预后较差。近20年来，得益于高强度化疗、造血干细胞移植、新型小分子靶向药物、嵌合抗原受体T细胞（CAR-T细胞）等治疗的进展，血液病患者的预后得到改善，生存期明显延长。然而，严重并发症的发生率也相应增加，血液病重症患者越来越多，尤其是伴有基础疾病的中老年患者。所以，需要提高血液专科医师诊疗水平、提高重症患者救治成功率，使患者渡过危险期，为后续治疗创造机会，最终改善血液病患者预后。

目前国内床位数超过200张的血液病中心越来越多，按3%～5%的重症监护病房（Intensive care unit, ICU）床位配置要求，每个中心需要10张左右ICU床位，来集中管理血液病重症患者。并且需要具备处理严重并发症、脏器功能支持及血液原发病治疗的综合能力，大部分医院的综合ICU往往不能满足血液科这种需求，血液病中心迫切需要建立血液重症监护病房（Hematology intensive care unit, HCU）。《血液专科重症单元危重症患者诊疗和管理中国专家共识（2025版）》[1]（简称《血液重症共识》），由胡豫教授牵头，联合领域内权威专家，参考国内HCU运行经验和相关指南共同完成。《血液重症共识》从多个方面提出了具体建议，将为规范血液病重症患者的诊疗和管理提供指导。本文重点从HCU的运行模式、主要收治对象以及血液病重症患者病情评估与治疗三个方面对这篇共识做一述评，并结合国内多家中心HCU建设情况谈一些体会。

一、建立HCU的必要性

血液科重症患者比例高、病情重、预后差，在处理严重并发症或器官功能支持的同时，需兼顾原发血液病的治疗。此外，重症患者的管理非常牵扯临床医师的精力，重症患者集中管理，减轻医疗组和病房值班医师的负担和压力，并且减少医疗纠纷。腾出的精力和时间利于临床研究等科研工作的开展，HCU的建立有利于学科建设和发展。HCU设置在血液内科病区内，便于重症患者及时转诊，便于加强与普通病房主管医师之间协作救治。所以，设置HCU重要且必要，《血液重症共识》的发布非常及时，将指导我国HCU的建设进程。

二、国内HCU的发展和运行模式

国外（如欧洲、韩国等）早已积累HCU经验，我国HCU虽然处于起步阶段，但近几年发展迅速。2011年华中科技大学同济医学院附属协和医院率先成立了HCU，随后哈尔滨医科大学附属第一医院（2017年）、苏州大学附属第一医院（2020年）以及中国医学科学院血液病医院（2022年）等多家医院血液科陆续成立HCU。由于不同中心医疗条件存在差异（如医疗布局规划、医疗设备、人员配置或团队培训等），其HCU运行模式也不尽相同。共识专家组提出了两种HCU运行模式，即ICU模式和pre-ICU模式，见表1。其中ICU模式是目前国内多数中心的运行模式，pre-ICU模式是《血液重症共识》中重点介绍的模式。

**表1:** 血液重症监护病房（HCU）两种运行模式的特征比较

特征	ICU模式	pre-ICU模式
运行模式	综合ICU内设有血液亚专科床位或血液重症医疗组，亦可设置在血液病区内，病房建设、设备配置、运行模式和管理上与综合ICU基本一致	可设置在血液病区内，集中管理血液重症患者，综合ICU作为会诊科室辅助支持
主管医师	高级职称的血液专科医师与ICU专科医师协作管理	经ICU培训并获得相应资质的血液专科医师主导管理
优点	血液科医师与ICU专科医师发挥专业所长	更注重血液专科的特点
缺点	生命支持技术要求高，运行成本较高	生命支持技术依赖ICU会诊支持，硬件和团队配置可能不如ICU模式完善

**注** ICU：重症监护病房

哈尔滨医科大学附属第一医院HCU（哈医大一院HCU）是标准的血液专科pre-ICU模式，设置在血液病区内，是血液科的一个病区，病房配置和运行标准参考《ICU建设与管理相关指南》[2]–[4]。HCU的必要设备包括吊塔、心电监护仪、中央监护系统、呼吸机、转运呼吸机、无创呼吸机、连续性肾脏替代治疗（Continuous renal replacement therapy, CRRT）机、血浆置换机、纤维支气管镜、心肺复苏机、心电图机、血气分析仪、以及空气净化和紫外消毒设备或层流装置等。HCU医护有血液科和ICU学习或培训经历，可独立抢救危重症患者，全权负责诊治，转入重症患者的普通病房主管医师可参与协助救治。医师参加中华医学会重症医学专科资质培训班（简称“5C培训”项目）并获得重症专科资质，掌握重症常见操作技术，包括气管插管、中心静脉置管、CRRT、血浆置换、白细胞或红细胞分离单采、经鼻或气管导管电子支气管镜等技术。

我们推荐对于不能达到上述HCU配置的单位，亦可于血液科病区内设置抢救室，选派血液科医师接受重症医学学习和培训，熟悉重症医学诊治理念，关注重症风险高的血液病患者。做到规范化管理血液重症患者，及时识别及判断转入ICU的最佳时机，避免延迟到生命处于极度危险的情况时，丧失逆转机会。

无论哪种模式，均应具备共同的特点，即具备严重并发症救治、脏器功能支持以及血液原发病治疗的综合能力。但不同HCU医疗模式可能因治疗目标或专业领域差异产生潜在冲突，可通过建立标准化协作流程，制定血液重症跨学科协作规范，明确各科室职责，更好地衔接专科治疗与ICU的生命支持。

三、HCU主要收治对象

共识强调HCU并非综合ICU的分支，HCU收治患者可以包括血液科的危、重、急症患者。结合多中心HCU收治患者的特点，分类归纳如下。

1. 血液病相关的严重并发症患者：严重或致命性出血性疾病，如血小板减少性紫癜、初治急性早幼粒细胞白血病（Acute promyelocytic leukemia，APL）因弥散性血管内凝血（Disseminated intravascular coagulation，DIC）等，应立即收入HCU给予生命支持、止血和输血等治疗；此外出血风险较高情况，如溴敌隆中毒、华法林等抗凝药过量亦建议收入HCU给予特效解毒和急救处理，一般于48～72 h内转回普通病房。

重症感染，如脓毒症/脓毒性休克、中枢神经系统感染或重症肺感染伴呼吸衰竭等。其中，伴脓毒症的血液病患者是HCU主要收治对象之一，严重脓毒症或脓毒性休克患者应立即转入HCU；严重脓毒症高风险患者，如国家早期预警评分（NEWS）≥7分或quick-序贯器官衰竭评分（SOFA）≥2分、高乳酸血症、严重代谢性酸中毒、中性粒细胞缺乏（粒缺）期≥7 d、老年等建议尽早转入HCU；中风险患者（NEWS评分4～6分）应持续评估，病情进展尽早转入HCU。

粒缺期脓毒症/脓毒性休克较为凶险。哈医大一院HCU回顾性分析了2017–2024年245例伴脓毒症血液病患者（约占HCU患者的20%），其中粒缺脓毒症88例（35.91%），主要见于急性白血病化疗后（49/88，55.68%）。转入HCU时，对比非粒缺脓毒症，粒缺脓毒症多因脓毒性休克转入［53.40%（47/88）对36.94%（58/157），*P*<0.05］，死亡率更高［46.59%（41/88）对32.48%（51/157），*P*<0.05］[5]。

初诊急性髓系白血病（Acute myelogenous leukemia, AML）容易出现感染、出血、白细胞瘀滞、DIC等严重并发症，对HCU的需求逐年升高。苏州医科大学附属第一医院HCU（苏医大一院HCU）回顾性分析91例初诊AML患者的临床特征，其中38.5%患者早期死亡，入住HCU的主要原因为肺部感染（78.0%），其次分别是呼吸衰竭（44.0%）、肝功能不全（28.6%）、肾功能不全（27.5%）、DIC（25.3%）和脓毒症（23.1%）。DIC、脓毒症及心功能不全是患者28 d死亡的独立危险因素[6]。

初治APL对诱导分化治疗敏感度高，如能获得完全缓解，多数患者可实现长期无病生存。诱导缓解治疗前或治疗期间出现严重并发症有早期死亡风险的患者，是HCU中需重点关注对象。哈医大一院HCU回顾分析44例初治APL重症患者临床特征[7]，脑出血是最常见的转入原因（20例，45.45%），亦是最重要的死亡病因（18例，40.90%）；其次为肺出血（8例，18.18%）、脓毒症或脓毒性休克（7例，15.9%）；因严重血栓并发症或分化综合征（Differentiation syndrome, DS）转入的患者少见（各1例）。苏医大一院HCU回顾性分析32例初诊APL患者的临床资料[8]，多因素分析显示诱导化疗后继发弥漫性肺泡出血（Diffuse alveolar hemorrhage, DAH）是患者预后不良的独立危险因素（*HR*＝23.8，*P*＝0.027，95%*CI*：1.437～327.439），9例DAH患者于HCU中经诱导治疗为基础的重症支持治疗均好转。

2. 血液病治疗相关严重并发症患者：如APL诱导分化中出现严重DS伴呼吸困难，肿瘤溶解综合征伴高钾血症或急性肾损伤，造血干细胞移植后出现急性移植物抗宿主病、植入综合征，以及CAR-T细胞免疫治疗或造血干细胞移植后出现3～4级细胞因子释放综合征（Cytokine release syndrome，CRS）伴休克等；因药物不良反应的重症患者，如高甲氨蝶呤血症导致骨髓抑制、皮肤黏膜损伤、重症感染或肾毒性的重症患者，CRRT可有效降低甲氨蝶呤浓度。一旦识别严重并发症且需器官功能支持的患者，均应及时转入HCU。

3. 进展迅速且死亡率高的血液病患者：如噬血细胞综合征、高白细胞急性白血病、白细胞明显升高的初治APL、血栓性血小板减少性紫癜（Thrombotic thrombocytopenic purpura，TTP）等。其中，TTP是罕见且危及生命急危重症，随着急诊科、神经内科以及基层医院对TTP早期识别提高，HCU中TTP亦常见。建议确诊或高度怀疑TTP患者于HCU中启动紧急治疗，如病情持续稳定，可考虑转回普通病房。

华中科技大学同济医学院附属协和医院HCU分析79例获得性TTP患者特点[9]，患者均起病急，多表现为血小板减少（96.2%）、微血管病性溶血性贫血（93.1%）、神经症状（89.6%）的三联征，其中14例（17.7%）患者死亡，尽早入住HCU进行重症监测及器官支持，同时启动血浆置换、糖皮质激素及利妥昔单抗等治疗可降低死亡率，改善预后。

4. 需器官功能支持或血液净化治疗的血液病患者：通气功能障碍（PaCO_2_明显升高、pH<7.25），或传统氧疗无法改善的低氧血症，尽早入HCU优先使用经鼻高流量湿化氧疗（HFNC）改善氧合，有利于减少气管插管；持续血流动力学受损（收缩压小于90 mmHg，1 mmHg＝0.133 kPa）尽早入HCU。需血液净化治疗的血液病患者，如多发性骨髓瘤伴肾功能衰竭、肿瘤溶解综合征、高甲氨蝶呤血症、代谢性酸中毒、高钾血症等接受CRRT治疗；急性白血病高白细胞瘀滞患者接受血细胞单采预处理；TTP、噬血细胞综合征伴肝功能衰竭和凝血异常、高黏滞综合征、严重的自身免疫性溶血性贫血等接受治疗性血浆置换。病情稳定的患者经血液净化治疗后可转回普通病房。

5. 其他：除上述主要收治对象外，还包括一部分需要生命支持和监护的血液重症患者，如心脏骤停或猝死，输血或药物治疗导致的过敏性休克，基础疾病严重并发症（酮症酸中毒、恶性心律失常、快速房颤伴低血压等），严重内环境紊乱（乳酸酸中毒、严重高钾血症等）。

四、血液病重症患者病情评估与治疗

血液病重症患者病情评估非常重要，目的在于早期发现高危或有生命危险的重症患者，及时转入HCU，避免错过最佳抢救时机。我们建议病情评估除基础生命体征、专科查体和实验室检查外，还应重视量化评分工具的应用，中国医学科学院血液病医院HCU探讨应用SOFA评分及其动态变化（ΔSOFA）预测死亡率的价值[10]，发现第1～3天SOFA评分和第1天ΔSOFA与患者在HCU的死亡率显著相关，在预测HCU中死亡率具有一定价值。在评估病情时不应延误治疗，包括严重并发症的处置、器官功能支持以及原发血液病治疗。

粒缺伴发热的恶性血液病患者常见，且易进展为严重脓毒症或脓毒性休克。临床医师首先通过询问病史、体格检查、实验室及影像学等辅助检查判断患者是否为感染性发热，明确感染灶、致病病原体，同时评估原发病状态如恶性血液病是否达完全缓解，粒缺持续时间和抗生素耐药危险等；应用多国癌症支持治疗协会（Multinational association of supportive care in cancer, MASCC）风险评分系统分层管理粒缺伴发热患者，评估是否需要住院静脉滴注抗生素治疗[11]；应用SOFA和quick-SOFA评分早期识别或预测脓毒症。因脓毒症或脓毒性休克转入HCU的患者，启动早期集束化治疗[12]。同时评估病情严重程度和死亡风险，可应用SOFA和APACHE Ⅱ等标准评分系统；亦应关注影响不良预后的独立危险因素，如粒缺持续时间大于7 d[13]、病原体耐药性、高龄、慢性基础疾病（如糖尿病、肾脏疾病和癌症晚期等）等。其他治疗包括调整或暂停原发病化疗方案、感染源控制、炎症与免疫调理、G-CSF、血糖控制、镇静镇痛、应激性溃疡预防、输血、营养支持，以及器官功能支持如CRRT或机械通气等。

HCU中约20%患者因呼吸困难转入，临床医师首先判断呼吸衰竭类型和病因，评估患者呼吸频率、动脉血氧分压、氧合指数以及心功能等指标。常见病因包括重症肺感染，急性心力衰竭、DAH、CAR-T等免疫治疗相关CRS导致的急性呼吸窘迫综合征（Acute respiratory distress syndrome, ARDS）；高白细胞急性白血病伴呼吸困难，或APL诱导分化过程中DS继发呼吸衰竭；多发性骨髓瘤因肾功不全前负荷过重继发呼吸衰竭等。评估患者是否需转入HCU，主要取决于是否需要呼吸监测，或是否需要HFNC或机械通气治疗。强调早期评估，避免延迟至危重症转入错失最佳抢救时机。比如伴轻中度Ⅰ型呼吸衰竭（100 mmHg ≤ PaO_2_/FiO_2_ < 300 mmHg），或轻度呼吸窘迫（呼吸频率> 24次/min），或轻度通气功能障碍，或对传统氧疗或无创通气不耐受或有禁忌证者，在进展为危重症前早期转入HCU优先使用HFNC迅速改善氧合、减少气管插管[14]。而对于心跳呼吸骤停，或自主呼吸微弱、昏迷，或极重度Ⅰ型呼吸衰竭（PaO_2_/FiO_2_< 60 mmHg），或通气功能障碍（PaCO_2_明显升高、pH<7.25）的患者，应迅速转入HCU紧急气管插管有创机械通气；如确诊或怀疑ARDS应转入HCU，治疗原则主要是积极病因治疗，呼吸支持治疗以及抗炎、利尿等。

恶性血液病重症患者的原发病治疗应个体化，共识推荐“三级分层治疗框架”，通过综合评估疾病状态（如恶性血液病类型、初诊或复发），器官功能以及治疗耐受性等因素。分为推荐治疗、慎重治疗和不建议治疗三类。强调动态评估原则，对病情不稳定的患者采取“观察-调整”策略，待临床状况改善后再考虑原发病治疗。如伴粒缺脓毒症的AML患者，应考虑暂停化疗或调整方案，待感染控制病情稳定后继续标准方案化疗；对于伴粒缺脓毒症初治APL患者，经综合评估病情后，亦可考虑同时诱导分化治疗。另外，应重视患者及家属治疗意愿，体现了生物-心理-社会医学模式在血液重症领域的重要性。

HCU自成立以来已逐步形成了一套标准化、规范化的管理体系。然而，当前血液重症诸多研究领域仍处于初步阶段，尚未形成广泛共识，呼吁同行加强合作，分享最新研究进展，共同探索血液重症的诊疗新模式，提高空白领域的循证等级，达成诊疗的共识，为血液病患者提供更优质的医疗服务。

五、小结

《血液重症共识》为国内首部针对血液重症患者诊疗和管理的专家共识，结合国内HCU一线管理经验，参考相关指南和专家组建议精心撰写该共识。共识中提出了诸多可行性建议，为计划筹建HCU的单位提供了经验借鉴。

血液病重症患者的救治能力是衡量血液科综合实力重要指标之一，从实际运行经验和效果来看，我们倡议具备条件的血液中心应积极筹建HCU。并期待通过学术交流和研讨，在HCU运行模式、血液病重症并发症管理以及重症患者原发病治疗等方面形成病房建设标准、制定临床路径、专家共识或指南，促进专科医疗精细化发展，同时培养更多高水平血液重症医务人员。

随着HCU广泛建立，血液重症患者救治综合能力不断提升，最终为血液病患者提供高质量的生命支持，切实降低重症血液病患者死亡率发挥关键作用。
